# Anti-Inflammatory Activity of *Lactobacillus* on Carrageenan-Induced Paw Edema in Male Wistar Rats

**DOI:** 10.1155/2012/752015

**Published:** 2012-02-22

**Authors:** Sarika Amdekar, Purabi Roy, Vinod Singh, Avnish Kumar, Rambir Singh, Poonam Sharma

**Affiliations:** ^1^Department of Microbiology, Barkatullah University, Bhopal 462026, India; ^2^Department of Biotechnology, Dr. B. R. Ambedkar University, Agra, 282004, India; ^3^Institute of Biomedical Sciences, Bundelkhand University, Jhansi 284001, India; ^4^Department of Zoology, Institute of Basic Sciences, Bundelkhand University, Jhansi, 284001, India

## Abstract

*Introduction*. *Lactobacillus casei* and *Lactobacillus acidophilus* were used to assess the anti-inflammatory properties in carrageenan induced acute inflammatory model. *Materials and Methods*. Diclofenac sodium was used as standard drug at concentration of 150 mg/kg of body weight. Culture of *Lactobacillus*  2 × 10^7^ CFU/ml was given orally. Edema was induced with 1% carrageenan to all the groups after one hour of the oral treatments. Paw thickness was checked at *t* = 1, 2, 3, 4, 5, and 24 hours. Stair climbing score and motility score were assessed at *t* = 24 hours. Cytokines assay for IL-6, IL-10, and TNF-*α* was performed on serum samples. *Results*. *Lactobacillus* showed a statistically significant decrease in paw thickness at *P* < 0.001. *L. acidophilus* and *L. casei* decreased by 32% and 28% in paw thickness. They both significantly increased the stair climbing and motility score. *Lactobacillus* treatment significantly downregulated IL-6 and TNF-*α* while upregulated IL-10 at *P* < 0.0001. *Conclusion*. *L. casei* and *L. acidophilus* significantly decreased the inflammatory reactions induced by carrageenan. This study has also proposed that *Lactobacillus* ameliorated the inflammatory reaction by downregulating the proinflammatory cytokines pathway.

## 1. Introduction

Inflammation is a local response of living mammalian tissues to injury. It is a body defense reaction in order to eliminate or limit the spread of injurious agent [1]. There are various components to an inflammatory reaction that can contribute to the associated symptoms and tissue injury. Edema, leukocyte infiltration, and granuloma formation represent such components of inflammation. Though, it is a defense mechanism. The complex events and mediators involved in the inflammatory reaction can induce or aggravate many reactions [2]. 

 As it is well known, the probiotic bacteria are nonpathogenic and consumed as/with food since a long time. The use of probiotic bacteria in dietary supplements or dietary products is widely documented in the literature. *Lactobacillus* and its important species like *L. casei* and *L. acidophilus* have been used against many pathological and disease conditions. *Lactobacillus* is known for its antimicrobial, antilipidemic, immunomodulatory, anticancerous, antidiabetic, and antiarthritic properties [3–10]. *Lactobacillus* has been assessed for its immunomodulatory properties in different experiments [11–15].

According to the WHO report, about 70–80% of the world's population rely on nonconventional medicine mainly from herbal sources in their primary health care [16, 17]. Especially, its demand is increasing day by day in developing countries where the cost of consulting a physician and price of medicine are beyond the limit of most people [18]. These drugs are anti-inflammatory and used to ease pain in various conditions including: arthritis, muscle, and ligament pains. Conventional drugs treatments are limited in their effectiveness in managing the incidence and outcome of many inflammatory diseases. They also present a significant number of side effects in patients [19]. 

With these facts taken into account, present study was planned to find out the possibility of anti-inflammatory activity of *L. casei* and *L. acidophilus* using carrageenan-induced acute inflammatory model. 

## 2. Materials and Methods

### 2.1. Bacterial Cultures

*Lactobacillus acidophilus* (ATCC 314) and *Lactobacillus casei* (ATCC 334) were purchased from Hi Media, Navi Mumbai, India. Lyophilized culture was streaked over de Mann Rogosa Sharpe Agar (MRS) at 37°C in anaerobic condition.

### 2.2. Drugs and Chemicals Used

Carrageenan was purchased from Hi Media, Mumbai, India. Standard anti-inflammatory drug diclofenac sodium was purchased from Recon, Bangalore, India. Cytokines assay kits were purchased from Ray Biotech, Norcross GA, USA and DNA Bio, Hyderabad, Andhra Pradesh, India.

### 2.3. Animals

35 male Wistar rats (200 gm each) were used for the present study. They were fed with standard pellet diet and water *ad libitum*. All animals were acclimatized for at least one week before the experimental session. All the experimental procedures were done following the guidelines of the Institutional Animals Ethics Committee (IEAC).

### 2.4. Evaluation of Anti-Inflammatory Activity

For the anti-inflammatory activity against the acute inflammation, animals were divided into five groups.

Group A (carrageenan control) did not receive any oral treatment; Group B (control) received 500 *μ*L of distilled water; Group C and Group D received 2 × 10^7^ CFU/mL of *L. casei* and *L. acidophilus*, respectively, suspended in 500 *μ*L of distilled water. Group E (positive control) animals were administered with diclofenac sodium (150 mg/Kg body weight).

### 2.5. Carrageenan-Induced Acute Inflammatory Model

Anti-inflammatory activity was measured using carrageenan-induced rat paw edema assay [20, 21]. Edema was induced by subplantar injection of 100 *μ*L of 1% freshly prepared solution of carrageenan in distilled water into the right-hind paws of each rat of all the groups except the group A. Animals of group B/C, D/E were treated with the single dose of vehicle, cultures, and drug, respectively; 30 minutes prior to carrageenan injection. Paw thickness were measured just before the carrageenan injection, that is, at “0 hour” and then at 1, 2, 3, 4, and 24th hour after carrageenan injection. Increase in paw thickness was measured as the difference in paw thickness at “0 hour” and paw thickness at respective hours.

### 2.6. Stair Climbing Activity Test

Overnight fasting animals were trained for one week to climb a staircase with steps at 5, 10, and 15 cm having water at the second and food at the third step. Climbing ability of the rats in above groups was scored 0 if the rats did not climb; 1, if the rats climbed onto step 1; 2, if the rats climbed onto step 2; 3, if the rat could climb all the three steps [22, 23].

### 2.7. Motility Test

The motility pattern of the rats was observed for a period of 5 minutes and scored 0, if the rat walked with difficulty and avoided touching the toes of the inflamed paw to the floor; 1, if the rat walked with little difficulty, but with toe touching the floor; 2, if the rat walked easily [22, 23].

### 2.8. Sample Collection

After 24 hours, all animals were sacrificed with cardiovascular bleeding with the help of Di ethyl ether according to the guidelines of CPSCEA committee. Blood was collected for the cytokines assay. Blood samples were left to coagulate for room temperature for 60 minutes and then centrifuged at 1500 g for 15 minutes and crude serum was kept in new tubes. Serum was harvested and stored at −20°C until use.

### 2.9. Cytokines Assay

IL-10 (anti-inflammatory cytokines) and IL-6, TNF-*α* (proinflammatory cytokines) in picogram per millilitre (pg/mL) were estimated with the help of ELISA Reader (Lisa Plus, Germany). Serum samples were used. IL-6 and TNF-*α* (Ray Bio ) and IL-10 (DNA bio) ELISA kits were used. Assays were performed according to the manufacturer's recommendations.

### 2.10. Statistical Analysis

The value for edema volume is expressed as mean *±* SEM of seven observations and ANOVA followed by post hoc test. Duncans test was used to compare the groups. The stair climbing ability test and motility are expressed as median scores and the Kruskal-Wallis test was used to compare the groups.

## 3. Results

### 3.1. Carrageenan-Induced Inflammation

**Figure 1:**
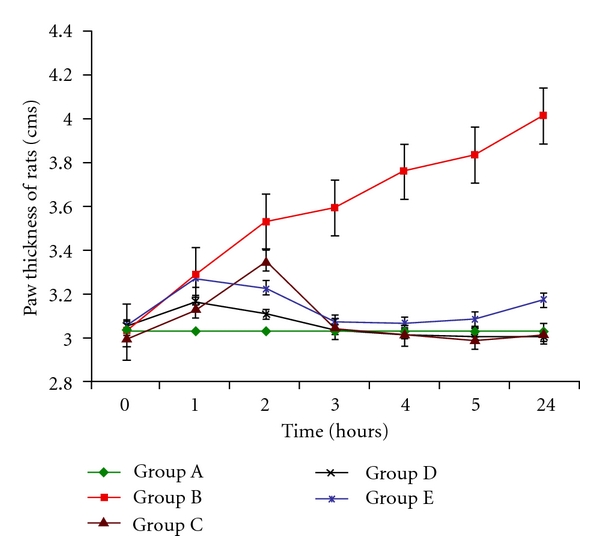
Change in paw thickness (cm) at *t* = 0,1, 2,3, 4,5, and 24 hours. *n* = 5 (significant at *P* < 0.001). Edema was induced by injecting 0.1 mL of 1% solution of carrageenan into the sub plantar surface of right-hind paw. Data are expressed as mean ± standard error of seven rats per group. Group A: carrageenan control; Group B: control; Group C: *L. casei* fed; Group D: *L. acidophilus*; Group E: positive control.

Injection of carrageenan into the hind paw induced a progressive edema reaching its maximum at 4 hours. In case of Group A animals paw thickness found at *t* = 0 was 3.023 ± 0.0408 cm and this remains constant at the end of 24 hours. Group B animals had showed an increase in paw thickness at each hour which was significant at *P* < 0.001. At 0 hours the thickness was 3.028 ± 0.040 cm, which increased to 3.59 ± 0.049 cm at *t* = 3 hours. At 24 hours the thickness was found to be 4.01 ± 0.025 cm. The paw thickness of Group C animals was 3 ± 0.028 cm which showed a mild increase at the end of 2nd hour, that is, 3.35 ± 0.0102 cm. After the 2nd hour it decreased to 3.04 ± 0.077 cm, 3.014 ± 0.0489 cm, 2.99 ± 0.0053 cm, and 3.014 ± 0.024 cm at the end of 3, 4, 5, and 24 hours, respectively. Group D animals showed an increase up to the 2nd hour. 3.03 ± 0.065 cm thickness was found at the end of third hour, which decreased to 3.0014 ± 0.0024 cm at *t* = 24 hours. So, Groups C and D indicated a statistically significant decrease in paw thickness (*P* < 0.001). Group E animals also showed an increase in paw thickness of 3.042 ± 0.077 cm (*t* = 0 hours), 3.26 ± 0.069 cm (*t* = 1 hours), 3.22 ± 0.0184 cm (*t* = 2 hours), and 3.074 ± 0.0762 cm (*t* = 3 hours). It increased after the third hour to 3.174 ± 0.057 cm (*t* = 24 hours). These values were found to be statistically significant at *P* < 0.001 (Figure 1). 

### 3.2. Stair Climbing Activity

**Figure 2:**
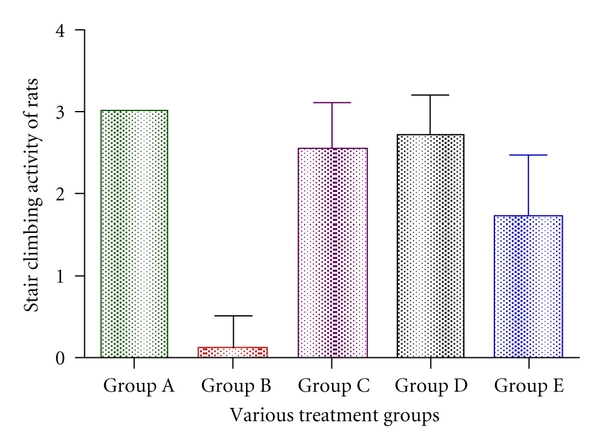
Effect of *Lactobacillus* on impairment in stair climbing activity score associated carrageenan-induced inflammation. Edema was induced by injecting 0.1 mL of 1% solution of carrageenan into subplantar surface of right-hind paw. The drugs were administered subcutaneously 30 minutes before injecting inflammagen. Stair climbing activity was observed at the time of peak inflammation (4 hours for carrageenan). Data are expressed as mean ± standard error of seven rats per group. Group A: carrageenan control; Group B: control; Group C: *L. casei* fed; Group D: *L. acidophilus*; Group E: positive control.

Hyperalgesia was induced by carrageenan in Group B rats. Their stair climbing activity was 0.28 ± 0.244, while highest score was observed in Group A animals at  3 ± 0.00  (statistically significant; *P* < 0.001). *Lactobacillus *treatment significantly increased the stair climbing score of 2.57 ± 0.489 and 2.71 ± 0.408 for Group C and Group D respectively (significant at *P* < 0.001). Group E animals were showing a score of 1.71 ± 0.6122. Group B animal score was lowest in comparison to Groups A, C, D, and E animals (Figure 2).

### 3.3. Motility

**Figure 3:**
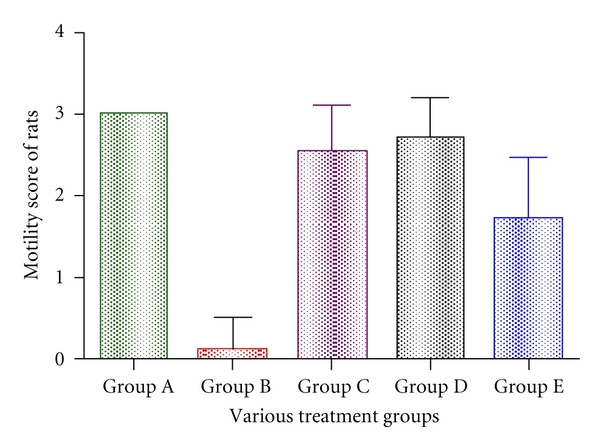
Effect of various drugs on impairment in motility associated with carrageenan-induced inflammation. Edema was induced by injecting 0.1 mL of 1% solution of carrageenan into subplantar surface of right-hind paw. The drugs were administered subcutaneously 30 minutes before injecting inflammagen. Motility score was observed at the time of peak inflammation (4 hours for carrageenan). Data are expressed as mean ± standard error of seven rats per group. Group A: carrageenan control; Group B: control; Group C: *L. casei* fed; Group D: *L. acidophilus*; Group E: positive control.

Walking ability of the rats to climb the staircase at the time of peak inflammation was checked by motility score. Group C and Group D animals were showing the highest score of 1.42 ± 0.489 (significant at *P* < 0.001). Score for Group A animals was  2 ± 0.00  whereas 0.14 ± 0.244 was found in case of group B animals. This was found to be the lowest when comparing Group E and Group B animals (Figure 3).

### 3.4. Cytokine Assay

Serum levels of IL-6 and TNF-*α* were highest in Group B animals, that is, 70.45 ± 0.266 pg/mL and 669.23 ± 0.050 pg/mL, respectively, while this group was showing lowest value of IL-10 (14.15 ± 0.035 pg/mL). On the contrary, *Lactobacillus* treatment statistically decreased the IL-6 and TNF-*α* in Group C and Group D at *P* < 0.0001. Lowest value for IL-6 was observed in case of Group D at 44.505 ± 0.198 pg/mL, while lowest value of TNF-*α* was observed for Group C at 420.77 ± 0.265 pg/mL. Group E animals was showing IL-6, TNF-*α*, and IL-10 values of 64.60 ± 0.131 pg/mL, 490.52 ± 0.228 pg/mL, and 29.675 ± 0.075 pg/mL, respectively. Only IL-6 concentration for Group E animals was not significant at *P* < 0.0001 while IL-10 and TNF-*α* concentrations were significant at *P* < 0.0001 (Table 1).

## 4. Discussion

Carrageenan-induced rat paw edema model is a suitable test for evaluating anti-inflammatory drugs, which has frequently been used to assess the antiedematous effect of the drug. Carrageenan is a strong chemical use for the release of inflammatory and proinflammatory mediators (prostaglandins, leukotrienes, histamine, bradykinin, TNF-*α*, etc.) [24].

The course of acute inflammation is biphasic. First phase starts with the release of histamine, serotonin, and kinins after the injection of phlogistic agent in the first few hours [25]. While the second phase is related to the release of prostaglandins like substances in 2-3 hours. Second phase is sensitive to both the clinically useful steroidal and nonsteroidal anti-inflammatory agent [26]. Prostaglandins are the main culprit responsible for acute inflammation. *Lactobacillus *sp might be containing some anti-inflammatory agent which is responsible for the blockage of prostaglandins and inflammatory pathway. 

In this model of inflammation, *L. casei* and *L. acidophilus* had very consistent anti-inflammatory activity and thus showed significant decrease in the paw thickness of rat (Group C and Group D). Previously, some researchers have also proposed the anti-inflammatory property of *Lactobacillus* in gut [27–29]. Although, the cyclooxygenase and lipoxygenase pathways play a pivotal role in the inflammatory process, the inhibition of cyclooxygenase is more effective in inhibiting carrageenan-induced inflammation than lipoxygenase inhibitors [30].

*Lactobacillus *might have inhibited the cyclooxygenase that synthesizes prostaglandins. Oral administration of *Lactobacillus *significantly downregulated the proinflammatory cytokines while upregulated the anti-inflammatory cytokines. Prostaglandins play essential role in inflammation [7]. Prostaglandins synthesis is down regulated by anti-inflammatory cytokines like IL-10, IL-4 and IL-13, which also check Coxygenase-2 synthesis. Coxygenase-2 is responsible for the increased production of prostaglandins. And hence, *L. casei* and *L. acidophilus* overcome the inflammation induced by carrageenan. Our present findings are consistent with the results of some other workers [7, 31–34]. Studies have shown that IL-10 has been found as a potent macrophage deactivator, which blocked TNF-*α*, IL-1, IL-6, IL-8, and GM-CSF by human monocytes [35].

Knowledge on the immune-mediated mechanisms in metabolic scenario has markedly increased in the recent past, evidencing the role that dietary components may have to modulate immunity by enhancing or suppressing the immune response. For instance, certain strains of probiotics have been demonstrated to be able to modulate the immune system by stimulating release of different patterns of cytokines by different cells. It could be able to modify the number of CD4 T cells and actively interfere with anti-inflammatory and proinflammatory signalling pathways by inducing production of IL-10 and reducing INF-*γ* and TNF-*α* release [36]. Probiotics modulate both innate and acquired immunity by interacting with indigenous bacteria and/or host mucosal cells to induce or modulate the immune response. This can be another possible reason behind its anti-inflammatory property in this study.

The present results suggest that *Lactobacillus* suppresses the first phase of carrageenan-induced paw edema, thus, confirming an NSAID-like property. The present study showed that *L. casei* and *L. acidophilus* have both analgesic and anti-inflammatory properties.

## Figures and Tables

**Table 1:** Effect of oral administration of *L. casei *and *L. acidophilus* on cytokines expression (pg/mL).

Groups	IL-6	TNF-*α*	IL-10
Group A	54.42 ± 0.193*	530.75 ± 0.337*	25.075 ± 0.047*
Group B	70.45 ± 0.266	669.23 ± 0.050	14.15 ± 0.035
Group C	44.57 ± 1.809*	420.77 ± 0.265*	35.65 ± 0.086*
Group D	44.50 ± 0.198*	425.77 ± 0.187*	38.725 ± 0.047*
Group E	64.60 ± 0.131*	490.52 ± 0.228*	29.675 ± 0.075*

Data are expressed as mean ± standard error of seven rats per group *Values along columns are statistically significant at *P* < 0.0001 when compared with Group B (control). Group A: carrageenan control; Group B: control; Group C: *L. casei* fed; Group D:* L. acidophilus*; Group E: positive control.
